# A transcriptome-wide association study identifies susceptibility genes for Parkinson’s disease

**DOI:** 10.1038/s41531-021-00221-7

**Published:** 2021-09-09

**Authors:** Shi Yao, Xi Zhang, Shu-Cheng Zou, Yong Zhu, Bo Li, Wei-Ping Kuang, Yan Guo, Xiao-Song Li, Liang Li, Xiao-Ye Wang

**Affiliations:** 1 Department of Neurosurgery, Hunan Brain Hospital, Clinical Medical School of Hunan University of Chinese Medicine, Changsha, Hunan P. R. China; 2 National and Local Joint Engineering Research Center of Biodiagnosis and Biotherapy, The Second Affiliated Hospital, Xi’an Jiaotong University, Xi’an, Shaanxi P. R. China; 3 Key Laboratory of Biomedical Information Engineering of Ministry of Education, Biomedical Informatics & Genomics Center, School of Life Science and Technology, Xi’an Jiaotong University, Xi’an, Shaanxi P. R. China; 4 Provincial Key Laboratory of TCM Diagnostics, Hunan University of Chinese Medicine, Changsha, Hunan P. R. China

**Keywords:** Parkinson's disease, Diagnostic markers, Genome-wide association studies

## Abstract

Genome-wide association study (GWAS) has seen great strides in revealing initial insights into the genetic architecture of Parkinson’s disease (PD). Since GWAS signals often reside in non-coding regions, relatively few of the associations have implicated specific biological mechanisms. Here, we aimed to integrate the GWAS results with large-scale expression quantitative trait loci (eQTL) in 13 brain tissues to identify candidate causal genes for PD. We conducted a transcriptome-wide association study (TWAS) for PD using the summary statistics of over 480,000 individuals from the most recent PD GWAS. We identified 18 genes significantly associated with PD after Bonferroni corrections. The most significant gene, *LRRC37A2*, was associated with PD in all 13 brain tissues, such as in the hypothalamus (*P* = 6.12 × 10^−22^) and nucleus accumbens basal ganglia (*P* = 5.62 × 10^−21^). We also identified eight conditionally independent genes, including four new genes at known PD loci: *CD38*, *LRRC37A2*, *RNF40*, and *ZSWIM7*. Through conditional analyses, we demonstrated that several of the GWAS significant signals on PD could be driven by genetically regulated gene expression. The most significant TWAS gene *LRRC37A2* accounts for 0.855 of the GWAS signal at its loci, and *ZSWIM7* accounts for all the GWAS signals at its loci. We further identified several phenotypes previously associated with PD by querying the single nucleotide polymorphisms (SNPs) in the final model of the identified genes in phenome databases. In conclusion, we prioritized genes that are likely to affect PD by using a TWAS approach and identified phenotypes associated with PD.

## Introduction

Parkinson’s disease (PD) is the second most common age-related neurodegenerative disorder after Alzheimer’s disease, characterized by the loss of nigrostriatal neurons in the substantia nigra^1^. PD is more common in the elderly, and the prevalence increased from 1% in people over 60 years to 3–4% in those over 80 years^2^. Based on twin and family studies, the heritability of PD has been estimated to be at least 27% and up to 60%^3,4^, suggesting a substantial involvement of genetic factors drives the phenotypic variance. Genome-wide association study (GWAS) has seen great strides and invaluable utilities in revealing initial insights into PD’s genetic architecture. The largest-to-date GWAS for PD identified 90 independent genome-wide significant association signals, including 38 novel risk variants^5^. Together, associated loci represent only a small fraction of PD’s genetic etiology, leaving a substantial proportion of genetic risk factors uncharacterized^6^.

Most disease- and trait-associated variants mapped through GWAS lie within non-coding regions and are not in linkage disequilibrium with any nonsynonymous coding single nucleotide polymorphisms (SNPs)^7,8^. Thus, the causal variants and target susceptible genes of most GWAS risk loci have not been identified^9^. Some studies, including the most recently published PD GWAS, have linked the GWAS risk loci to the nearest gene, which will inevitably lead to biases against longer genes and may not accurately depict the locus’s real effect. Many of these genetic variants are located in the gene regulatory elements^10,11^ and have hypothesized to influence complex traits by modulating gene regulatory programs, an intermediate between genetic variation and complex disease. Expression quantitative trait locus (eQTL) analysis seeks to identify genetic variants that affect the gene expression; several studies have successfully used this approach to identify putative susceptibility genes at GWAS risk loci for PD^12^. The enrichment of eQTLs of trait-associated variants also showed the importance of gene expression regulation^13,14^.

Recent studies have reported that regulatory variants may account for a large proportion of disease heritability that has not yet been identified through GWAS^15^. Many of these variants may have modest effect sizes, and it is difficult to identify them via a typical individual SNP-based GWAS study, even with a considerable sample size. Transcriptome-wide association studies (TWAS) that systematically investigate the relationship between genetically predicted gene expression and disease risk, providing a powerful approach to identify disease risk genes and uncovering possible causal genes at loci identified previously by GWAS^16–20^. Considering the essential role of epigenetic features in predicting gene expression, we developed an epigenetic element-based TWAS^21^. Briefly, for a given gene, we used eQTL data to impute the total expression across a large cohort of genotyped individuals with the epigenetic features as prior, followed by a test of association with disease risk. Compared with the single SNP-based GWAS study, TWAS can increase power in identifying disease-related genes, either by reducing the burden of multiple comparisons or by aggregating multiple expression-altering variants into a single test.

In the current study, we carried out a TWAS to prioritize candidate PD genes and to better understand the primary mechanisms that underlie PD genetic risk factors using the largest PD cohort currently available with 15,056 PD cases, 18,618 proxies, and 449,056 controls. We prioritized 18 genes whose predicted expression is significantly associated with PD risk, comprising 67 associations, after Bonferroni corrections. Through conditional analyses, we demonstrated that several of the GWAS significant signals on PD could be driven by genetically regulated gene expression. We identified eight conditionally independent genes through conditional analyses, including four new genes at known PD loci. We identified several phenotypes previously associated with PD by querying the SNPs in the final models of identified genes in phenome databases. Overall, our study suggests that a TWAS approach considering both genetic and epigenetic effects on gene expression is a powerful method to identify specific genes and mechanisms at each GWAS locus as determinants of PD risk.

## Results

### Transcriptome-wide association study

**Fig. 1:**
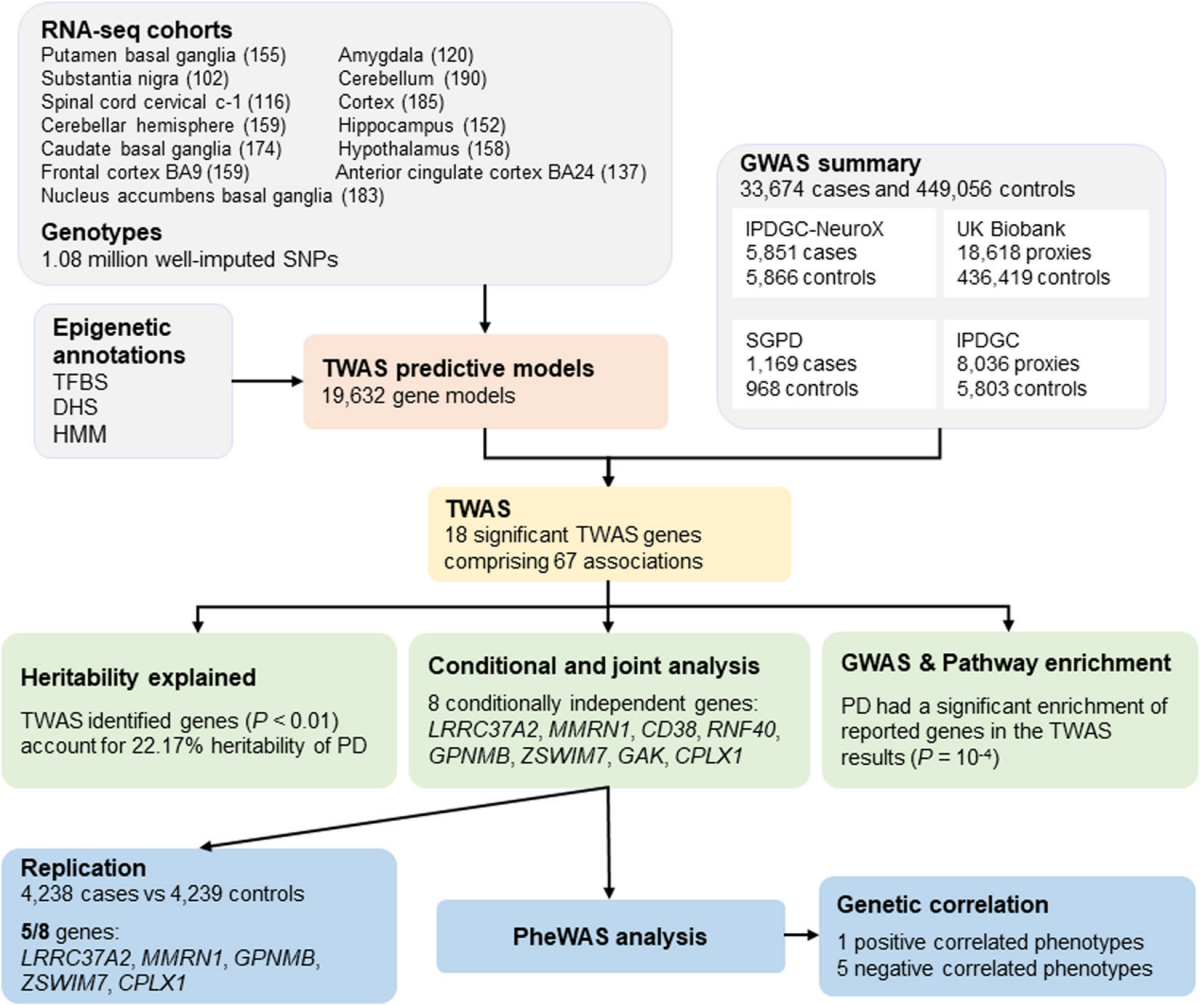
The workflow of the study. GWAS, genome-wide association study; TFBS, transcription factor binding site; DHS, DNase I hypersensitive sites; HMM, chromatin state segmentation by hidden Markov model; TWAS, transcriptome-wide association study; PD, Parkinson’s disease; PheWAS, phenome-wide association study.

**Fig. 2:**
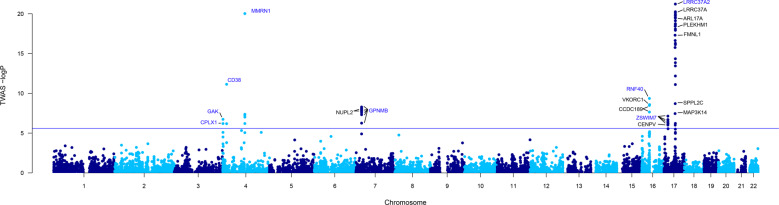
Manhattan plot of the TWAS results for PD. The blue line represents the Bonferroni-corrected significant thresholds, *P* = 2.55 × 10^−6^. Conditionally independent genes are listed in blue letters.

To identify genes associated with PD, we conducted a TWAS using summary data comprising 15,056 PD cases, 18,618 UK Biobank proxies, and 449,056 controls (Fig. 1). We used genotyping and transcriptome data from 13 brain tissues to build epigenetic-based gene expression prediction models. The tissue abbreviations and sample sizes are listed in Supplementary Table 1. We identified a total of 18 genes significantly associated with PD after Bonferroni corrections (*P* < 2.55 × 10^−6^, Fig. 2 and Supplementary Table 2), comprising 67 associations. The most significant gene, *LRRC37A2*, was associated with PD in all 13 tissues tested, such as the hypothalamus (*P* = 6.12 × 10^−22^) and the nucleus accumbens basal ganglia (*P* = 5.62 × 10^−21^). Amongst the signals, we found six genes only significant in one tissue, including *RNF40* (*P* = 4.42 × 10^−10^) and *CPLX1* (*P* = 6.36 × 10^−7^) in the cerebellar hemisphere, *VKORC1* in the cortex (*P* = 2.66 × 10^−9^), *MAP3K14* (*P* = 3.52 × 10^−8^) and *GAK* (*P* = 1.85 × 10^−7^) in the cerebellum, as well as *CENPV* in the frontal cortex BA9 (*P* = 6.07 × 10^−7^). Most of the genes (14/18) remained significant after permutation, suggesting that their signals were genuine and not due to chance.

### Conditionally testing GWAS signals

**Fig. 3:**
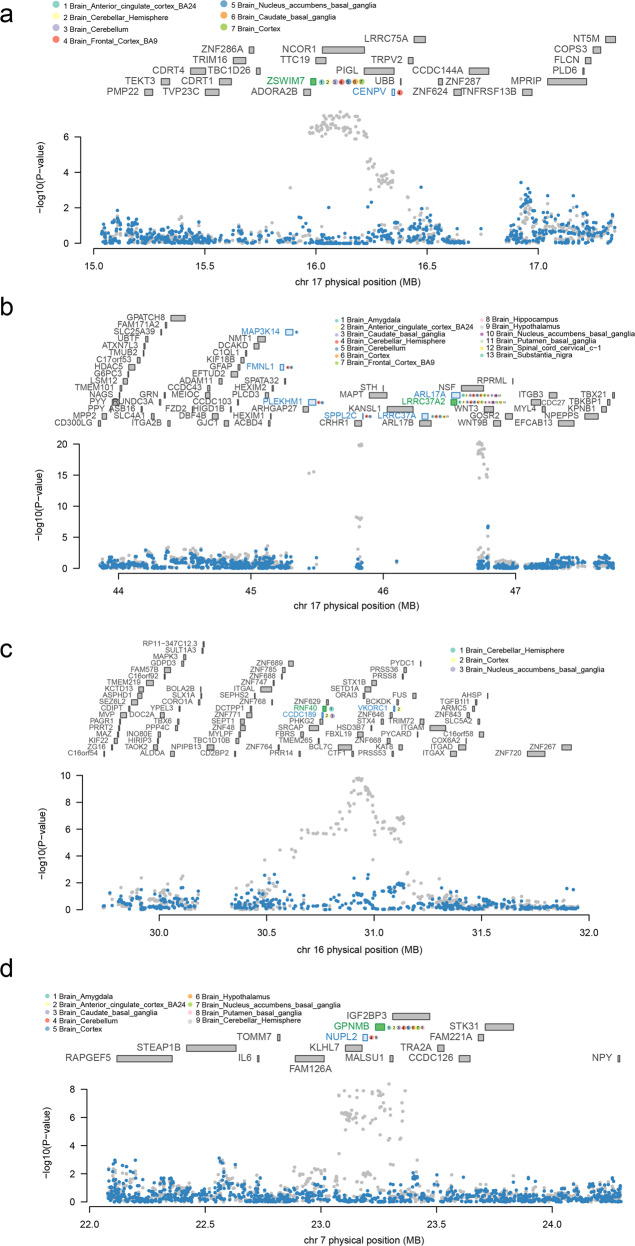
Regional association of TWAS hits. **a** Chromosome 17 regional association plot (part 1). **b** Chromosome 17 regional association plot (part 2). **c** Chromosome 16 regional association plot. **d** Chromosome 7 regional association plot. The marginally associated TWAS genes are shown in blue, and the conditionally significant genes are shown in green. The bottom panel shows a regional Manhattan plot of the GWAS data before (gray) and after (blue) conditioning on the green genes’ predicted expression.

Since most of the TWAS-identified genes overlapped with GWAS PD loci, we performed the conditional and joint analyses to check whether these signals were due to multiple associated features and how much GWAS signal remains after the gene expression is removed. We identified eight conditionally independent genes, including *LRRC37A2*, *MMRN1*, *CD38*, *RNF40*, *GPNMB*, *ZSWIM7*, *GAK*, and *CPLX1*. We observed that *ZSWIM7* accounts for all the signals at its loci (rs4566208 lead SNP_GWAS_ *P* = 3.90 × 10^−8^, conditioned on *ZSWIM7* lead SNP_GWAS_ *P* = 1) (Fig. 3a). Similarly, conditioning on *LRRC37A2* accounts for most of the signal at its loci (rs199452 lead SNP_GWAS_ *P* = 4.80 × 10^−21^, conditioned on *LRRC37A2* lead SNP_GWAS_ *P* = 0.17, accounting for 0.855 of the variances) (Fig. 3b). Conditioning on *RNF40* accounts for most of the loci’s variance (rs8050588 lead SNP_GWAS_ *P* = 1.60 × 10^−10^, conditioned on *RNF40* lead SNP_GWAS_ *P* = 0.066, accounting for 0.713 of the variances) (Fig. 3c). We also found that conditioned on the expression *GPNMB* accounts for 0.8 of the variances (rs466225 lead SNP_GWAS_ *P* = 4.30 × 10^−9^, conditioned on *GPNMB* lead SNP_GWAS_ *P* = 0.241) (Fig. 3d).

### Replication analyses

To assess our results’ robustness, we performed replication analyses to validate the conditionally independent genes. We found 46 reference models for these eight genes and five genes (*LRRC37A2*, *MMRN1*, *GPNMB*, *ZSWIM7*, *CPLX1*) significantly associated with PD in at least one brain tissue (*P* = 1.09 × 10^−3^, Supplementary Table 3). Similarly, *LRRC37A2* was associated with PD in all 13 tissues in the replication data, such as the hippocampus (*P* = 5.86 × 10^−11^) and the hypothalamus (*P* = 1.41 × 10^−10^).

### Partitioned heritability of TWAS-identified genes

We partitioned the heritability explained by SNPs around TWAS-identified genes and found that the genes identified by ten out of 13 tissues significantly contribute to PD’s heritability comparing to the percentage of SNPs (Table 1). For example, the genes identified in the brain cerebellum panel explained 5.52% (1.35%) of the estimated heritability, a 127.7× enrichment compared to the percentage of SNPs (*P* = 2.50 × 10^−5^). Partitioned heritability of TWAS genes in all the 13 tissues explained 22.17% of the estimated heritability, a 32.3× enrichment compared to the percentage of SNPs (*P* = 1.68 × 10^−5^).

**Table 1:** Partitioned heritability of TWAS-identified genes.

Tissue	SNPs Prop (%)	*h*^2^ Prop (%)	*h*^2^ Prop s.e. (%)	Enrichment	Enrichment s.e.	Enrichment *P*
AMY	0.08	1.35	3.77	17.5	49.0	0.750
ACC	0.03	2.41	0.69	71.4	20.5	**3.75** **×** **10**^**−4**^
CBG	0.07	2.78	1.67	39.8	23.8	0.104
CEH	0.07	8.11	2.49	123.8	38.0	**1.41** **×** **10**^**−3**^
CER	0.13	11.56	5.45	91.1	43.0	0.055
COR	0.04	5.52	1.35	127.7	31.2	**2.50** **×** **10**^**−5**^
FRO	0.13	5.08	1.21	38.6	9.2	**4.54** **×** **10**^**−6**^
HIP	0.09	3.69	1.58	42.0	18.0	**0.022**
HYP	0.07	3.25	1.62	46.1	23.0	**0.046**
NAB	0.05	3.90	1.19	73.6	22.5	**8.85** **×** **10**^**−4**^
PBG	0.05	5.83	1.84	127.3	40.1	**1.05** **×** **10**^**−3**^
SCC	0.07	2.78	1.29	39.7	18.4	**0.033**
SUB	0.02	3.72	2.04	160.8	88.0	0.067
Together	0.69	22.17	5.04	32.3	7.3	**1.68** **×** **10**^**−4**^

Bold values indicate significant *P* < 0.05. *HYP* brain hypothalamus, *NAB* brain nucleus accumbens basal ganglia, *ACC* brain anterior cingulate cortex BA24, *SCC* brain spinal cord cervical c-1, *HIP* brain hippocampus, *CEH* brain cerebellar hemisphere, *PBG* brain putamen basal ganglia, *COR* brain cortex, *CBG* brain caudate basal ganglia, *AMY* brain amygdala, *CER* brain cerebellum, *FRO* brain frontal cortex BA9, *SUB* brain substantia nigra.

### Gene set enrichment analyses

**Fig. 4:**
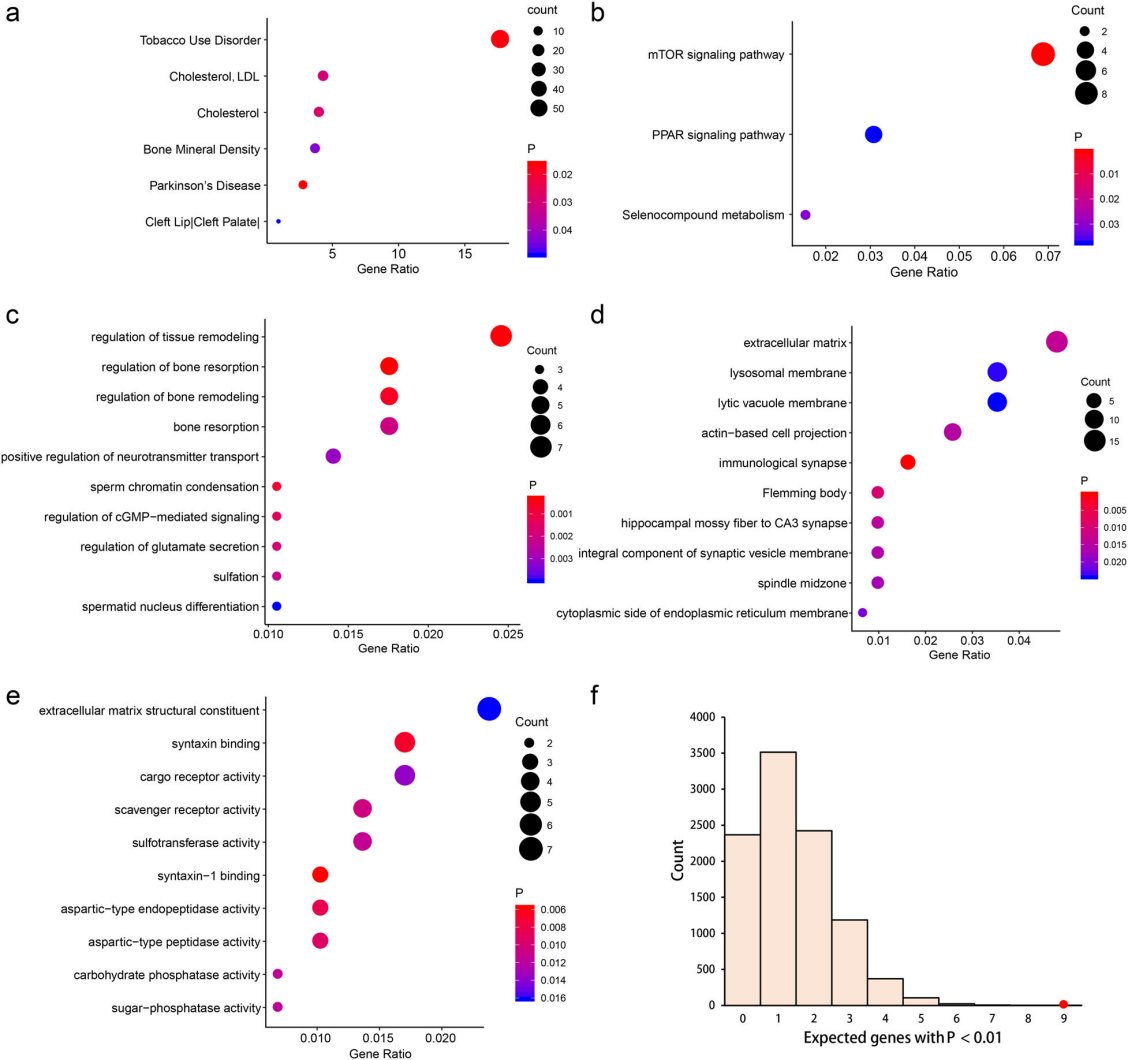
Pathway enrichment results of TWAS-identified genes. **a** GAD disease enrichment analyses of TWAS genes. **b** KEGG pathway enrichment analyses of TWAS genes. **c**–**e** Top ten GO terms enriched in TWAS genes, including biological processes (**c**), cellular component (**d**), and molecular function (**e**). **f** GWAS Catalog enrichment analyses of TWAS genes. The histogram shows the expected number of genes with *P* < 0.01 based on 10,000 random permutations. The large red point shows the observed number of previously known PD genes that fall below this threshold.

The Genetic Association Database (GAD) disease enrichment analyses detected six diseases (Fig. 4a), including PD (*P* = 0.011), tobacco use disorder (*P* = 0.012), cholesterol (*P* = 0.023), LDL cholesterol (*P* = 0.025), bone mineral density (*P* = 0.038), and cleft lip and cleft palate (*P* = 0.044). Three KEGG pathways were significantly enriched (Fig. 4b), including mTOR signaling pathway (*P* = 9.90 × 10^−4^), selenocompound metabolism (*P* = 0.031), as well as PPAR signaling pathway (*P* = 0.038). We also identified several GO terms enriched in TWAS-identified PD genes (Fig. 4c–e), such as regulation of bone resorption (biological processes, *P* = 2.87 × 10^−4^), immunological synapse (cellular component, *P* = 1.97 × 10^−4^), and syntaxin-1 binding (molecular function, *P* = 5.75 × 10^−3^).

### GWAS Catalog enrichment analyses

We found that nine TWAS-identified genes had been reported in the GWAS Catalog. The null expectation based on 10,000 randomly drawn gene sets proved that PD had a significant enrichment (*P* = 1.0 × 10^−4^) of GWAS Catalog-reported genes (Fig. 4f) in the TWAS results, which suggested that there are likely to be genuine disease associations among the genes that fail to meet strict genome-wide significance.

### Phenome-wide association studies

**Fig. 5:**
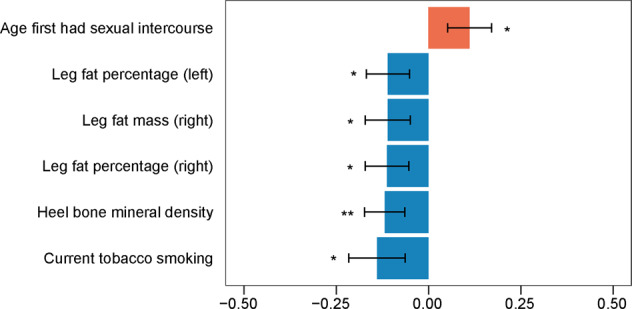
Genetic correlation between PD and phenotypes associated with top PD eQTLs. ^*^P < 0.05/122, ^**^P < 0.01/122, error bars indicate standard error of the genetic correlations.

We identified 166 phenotypes associated with the SNPs in the final model of the TWAS-identified conditionally independent genes, including activities such as neurological, psychiatric, and cognitive (Supplementary Fig. 1). We further conducted the genetic correlation between PD and 122 identified traits with currently available GWAS data to determine their relationship. The latest GWAS summary statistics are listed in Supplementary Table 4. We found that the age at first sexual intercourse positively correlated with PD (Fig. 5). We also reported five phenotypes negatively correlated with PD, including three impedance measures, heel bone mineral density, and current tobacco smoking (Fig. 5). Importantly, tobacco use disorder and bone mineral density have been identified through GAD disease enrichment analyses.

## Discussion

PD is a long-term movement disorder that affects approximately seven million people globally. Although recent GWAS has seen great strides in identifying risk loci associated with PD, the functional significance of these associations remains elusive. We conducted a PD TWAS using the summary statistics of over 480,000 individuals from the most recent PD GWAS. This approach creates genotype-expression reference panels using public consortia through Lasso and Elastic Net with epigenetic annotations as prior, allowing for imputation and association testing of independent large-scale data. We identified 18 genes associated with PD after Bonferroni corrections, comprising 67 associations localized to seven different regions in the genome.

Conditional and joint analyses identified eight independent genes and demonstrated that the TWAS expression signals were driving the significance for several previously implicated PD loci. For example, the most significant TWAS gene *LRRC37A2* accounts for 0.855 of the GWAS signal at its loci, and *ZSWIM7* accounts for all the GWAS signals at its loci. These results imply a limited residual association signal from the genetic variant in the GWAS locus after considering these predicted expression signals. Our identifications provide further support for three genes previously implicated by GWAS, whose expression was significantly associated with a possible causal change in PD risk by summary-based Mendelian randomization, including *MMRN1*, *GPNMB*, and *GAK*^5^. Another gene, *CPLX1*, was possibly associated with at least one QTL in public reference datasets but did not pass the Bonferroni corrections.

Additionally, our TWAS implicates four new genes at known PD loci, including *CD38*, *LRRC37A2*, *RNF40*, and *ZSWIM7*. For instance, *CD38* encodes the cluster of differentiation 38, also known as cyclic ADP ribose hydrolase, a glycoprotein on many immune cells’ surfaces. *CD38* strongly expressed in brain cells, including neurons, astrocytes, and microglial cells^22^. Of note, several data tend to indicate that *CD38* expression increase in the brain as a consequence of aging^23,24^, the primary risk factor associated with the vast majority of neurodegenerative diseases, including PD. Moreover, several experimental data demonstrated that *CD38* knockout mice are protected from neurodegenerative and neuroinflammatory insults^25,26^. Future studies could interrogate whether expression differences of other candidate genes are consistent with our findings.

In parallel to ours, Kia and colleagues^27^ conducted TWAS using expression weights from the CommonMind Consortium (CMC) dorsolateral prefrontal cortex to identify genes associated with PD. While considering the essential role of epigenetic features in predicting gene expression, our approach integrated the epigenetic information in the original TWAS to find gene–trait associations. Among the 18 genes we identified, eight genes (*MMRN1*, *CD38*, *NUPL2*, *GPNMB*, *RNF40*, *VKORC1*, *ZSWIM7*, and *CENPV*) were significantly heritable in CMC dorsolateral prefrontal cortex and qualified for the TWAS analyses. According to the published results, seven of the above eight genes associated with PD risk at an FDR level of 0.05 (Supplementary Table 5), including two genes (*CD38*, and *GPNMB*) with solid evidence for colocalization (PPH4 > 0.75)^27^. At the same time, numerous previously reported PD risk loci did not implicate in our TWAS. For example, Li and colleagues^28^ found the predicted gene expression of *SNCA* was associated with PD in peripheral monocytes, and published evidence suggested the association between *MAPT* expression and PD^29,30^. The most significant gene we identified, *LRRC37A2*, is located at the end of the *MAPT* locus, and the second most significant finding is *MMRN1* at the *SNCA* locus. However, *MAPT* and *SNCA* were not included in this project since they were not significantly heritable in any brain tissues at current sample sizes. Considering the previous evidence and the fact that we did not perform these associations in our project, we cannot determine the driver genes of PD at these loci, which can be regarded as one of the limitations of TWAS. Further studies with a larger sample size of reference data are needed to identify the driver genes of PB at these loci.

The GAD disease enrichment analyses of TWAS-identified genes detected six GAD diseases, including PD itself. Other enriched phenotypes, such as tobacco use disorder, cholesterol, and bone mineral density, also have been reported to be associated with PD. For example, tobacco use disorder, also known as nicotine dependence, is a chronic, relapsing disease defined as a compulsive craving to use it, despite harmful social consequences^31^. Epidemiological studies show that smoking is associated with a lower incidence of PD^32^. Moreover, nicotine stimulates striatal dopamine neurons that are damaged in PD and protect against neuronal insults in experimental models. Besides, previous researches have suggested that higher total- and LDL-cholesterol levels may be associated with lower risk and beneficial outcomes in PD^33,34^. Bone mineral density is the most widely used predictor for osteoporosis, and increasing evidence suggests that neurological conditions, including PD, are associated with an excess rate of osteoporosis and fracture risk^35^.

KEGG pathway enrichment analyses identified three pathways, including the mTOR signaling pathway, PPAR signaling pathway, and selenocompound metabolism. mTOR is a serine/threonine kinase that is the central component of mTORC1 and mTORC2 multiprotein complexes. mTOR regulates many integrated physiological functions of the nerve system, including neuronal development, synaptic plasticity, memory storage, and cognition^36^. The deregulation of mTOR signaling appears to be a common hallmark of human neurological disorders, including PD^37^, and mTORC1-induced transcripts enriched in a cluster of genes related to PD^38^. We also identified multiple GO terms that had been reported to be associated with PD. For example, above we have discussed the correlation between PD and osteoporosis, and three GO terms associated with osteoporosis were detected, such as regulation of bone resorption, regulation of bone remodeling, and bone resorption.

In summary, by using the TWAS method that we generated recently, we identified 18 genes associated with PD after Bonferroni corrections, comprising 67 associations localized to seven different regions in the genome. We identified eight conditionally independent genes, and we demonstrated that several of the GWAS significant signals on PD could be driven by genetically regulated gene expression. Our TWAS implicates four new genes at known PD loci: *CD38*, *LRRC37A2*, *RNF40*, and *ZSWIM7*. We further identified several phenotypes associated with PD by querying the SNPs in the final model of identified genes in phenome databases. In conclusion, we prioritized genes that are likely to affect PD by using a TWAS approach and identified phenotypes associated with PD.

## Methods

### GWAS summary statistics

#### Discovery GWAS data

We used the most recent GWAS summary statistics for PD. Details on participant ascertainment and quality control were previously reported by Nalls et al.^5^. The summary statistics comprising 15,056 PD cases, 18,618 UK Biobank proxy-cases (individuals who do not have PD but have a first-degree relative that does), and 449,056 controls. Since our approach depends on having dense summary-level data to overlap with the expression weights closely, we did not prune SNPs in the summary data.

#### Replicate GWAS data

Another GWAS summary statistic of PD, comprising 4238 PD cases and 4239 controls, was used as the replication data of this study. Details about ascertainment and quality control were previously reported by Pankratz et al.^39^. Similarly, all datasets employed standard UK Brain Bank criteria^40^ for the diagnosis of PD, with a modification to allow the inclusion of cases that had a family history of PD, since familial PD cases may have a more substantial genetic contribution than sporadic PD, making them potentially more informative for genetic studies.

### Transcriptome-wide association study

We used transcriptome and high-density genotyping data of European decedent from the Genotype-Tissue Expression (GTEx) study Pilot Project V8 (dbGap accession: phs000424.v8.p2) to establish gene expression prediction models^41^. In this project, we used genotyping and transcriptome data from 13 brain tissues to build epigenetic-based gene expression prediction models.

As described in our previously reported method^21^, we performed a TWAS using reference panels derived from tissue-specific gene expression coupled with genotypic data with the epigenetic features. For each gene *x*, we trained and evaluated the models for gene expression prediction in each round *y* of ten-fold cross-validation using the following steps. (a) We performed eQTL analyses with SNPs located within 1 Mb of the transcription start/end sites of the gene using the training data; (b) We then annotated the SNPs with epigenetic annotations. For each SNP, an epigenomic feature was labeled if the SNP overlapped with the feature. (c) We obtained multiple SNP sets according to the eQTL *P*-value threshold and epigenetic annotation. (d) For each SNP set *z*, we built an expression prediction model in the training dataset by using the Lasso and the Elastic Net (α = 0.5) methods as implemented in the glmnet R package. For each model, we evaluated its prediction performance by the coefficient of determination *R*^2^ between the predicted gene expression and the observed gene expression of the testing data, and averaged all the cross-validation data. For each gene *x*, the model with the highest mean *R*^2^ in the testing data was selected as the best model. Based on the parameters of the best model, we performed the eQTL analyses again using all the samples in the reference data and constructed each gene’s final prediction model. We estimated the associations between predicted expressions and PD with the combination of SNP-trait effect sizes while accounting for linkage disequilibrium among SNPs via Functional Summary-based Imputation (FUSION, http://gusevlab.org/projects/fusion/).

Since highly heritable genes were significantly enriched in trait associations^18^, we only focused on genes whose heritability did not overlap with zero with 95% confidence interval. We used a strict Bonferroni-corrected study-wise threshold with *P* = 2.55 × 10^−6^ (0.05/19,632, the total number of highly heritable genes across tissues). We applied the 1000 Genomes v3 LD panel for the TWAS. To assess the possibility of inflated association statistics from TWAS, we performed a permutation test. We shuffled the eQTL weights (*n* = 10,000) and recomputed an empirical association statistics conditional on the GWAS effects at the locus via FUSION.

### Conditional analyses

We performed conditional and joint analyses at each genome-wide Bonferroni-corrected TWAS genes to determine how much GWAS signal remains after the expression association from TWAS is removed. Moreover, for regions where TWAS identified multiple associated features, we jointly modeled these genes to determine the independent signals. Each PD GWAS association was conditioned on the joint gene model, one SNP at a time. We set the overlapping genes in the range of 1 Mb around each SNP, and the defined regions included only the transcribed region of the genes. We used the FUSION tool to perform the conditional and joint analyses with the *cis*-genetic component of expression we generated.

### Partitioned heritability estimation

The partitioned analysis is to quantify the heritability directly explained by SNPs in each functional category using the summary statistics compared with the null expectation, equal to the percentage of SNPs in the gene set. We estimated the partitioned heritability of PD by SNPs around TWAS-identified genes to see whether the identified genes significantly contribute to the PD heritability. Partitioned heritability of PD was estimated using LD score regression (LDSC) following the previously described methodology^42^. We partitioned the heritability explained by TWAS-identified genes with a less stringent threshold (*P* < 0.01) in each tissue by SNPs within 2 kb of the genes. We generated the LD score files using the open-source software available at https://github.com/bulik/ldsc/wiki/Partitioned-Heritability.

### Gene set enrichment analyses

To demonstrate TWAS’s ability to identify PD-related genes, we performed the GAD disease enrichment analyses via the Database for Annotation, Visualization, and Integrated Discovery (DAVID) tool (https://david.ncifcrf.gov/home.jsp) using default settings. A relaxed threshold of 0.01, rather than Bonferroni-correction, was used for GAD disease enrichment analyses since Bonferroni-correction assumes independence while genes tend to correlate due to co-expression. More genes will allow for better recapitulation and prioritization of appropriate pathways. We performed the GO and pathway enrichment analyses using the clusterProfiler R package. The GO terms (including biological processes, cellular components, and molecular functions) and pathways from KEGG (https://www.genome.jp/kegg/) were analyzed in this study.

### GWAS Catalog enrichment analyses

We tested whether the TWAS-identified genes enriched known PD-associated genes. The reported PD genes derived from the NHGRI GWAS Catalog^43^ identified using GWAS were regarded as the set of known PD-associated genes. We excluded studies that included the discovery dataset to make sure our known gene list was independent of the current analysis. We then counted the number of known disease-associated genes that had a TWAS *P*-value below 0.01. We compared this count to the null expectation based on 10,000 randomly drawn gene sets of similar size to the known disease gene set to derive an enrichment *P*-value.

### Phenome-wide association studies

To identify phenotypes that may be associated with PD, we conducted a phenome-wide association study (pheWAS) for each SNP in the final model of the identified genes. We reported the first five phenotypes (excluding PD) through public data provided by GWASAtlas (https://atlas.ctglab.nl) according to the *P*-values. We further conducted the genetic correlation between PD and identified traits with currently available GWAS data to determine their relationship. The analyses were carried out using LDSC at https://github.com/bulik/ldsc. The latest GWAS summary statistics were used for the correlation.

### Standard protocol approvals and participant consents

This study protocol was approved by the Ethics Committee of Hunan Brain Hospital. The study based on GWAS summary statistics does not require informed consent from all study participants. The methods were carried out in accordance with the approved guidelines.

### Reporting summary

Further information on research design is available in the Nature Research Reporting Summary linked to this article.

## Supplementary information

Supplementary information.

Reporting summary.

## Data Availability

The discovery GWAS summary statistic of PD was obtained from the link (https://bit.ly/2ofzGrk) shared by Nalls and colleagues^5^. The replicate GWAS summary statistic was obtained from the Genome-Wide Repository of Associations Between SNPs and Phenotypes database (https://grasp.nhlbi.nih.gov/FullResults.aspx). Other data that support the findings of this study are available from the corresponding author upon reasonable request.

## Code availability

The source code is available at https://github.com/studentyaoshi/etwas.
