# Supplementary material for: A transcriptome-wide association study identifies susceptibility genes for Parkinson’s disease
Source: NPJ Parkinsons Dis. 2021 Sep 9;7:79. doi: 10.1038/s41531-021-00221-7 (PMC8429416; doi:10.1038/s41531-021-00221-7)

Supplementary Table 1. Number of samples and significant heritable genes.

Tissue	Abbreviation	[a]Sample Size	[b]Highly heritable genes
Brain Amygdala	AMY	120	1,192
Brain Anterior cingulate cortex BA24	ACC	137	1,248
Brain Caudate basal ganglia	CBG	174	1,573
Brain Cerebellar hemisphere	CEH	159	2,010
Brain Cerebellum	CER	190	2,606
Brain Cortex	COR	185	1,769
Brain Frontal cortex BA9	FRO	159	1,549
Brain Hippocampus	HIP	152	1,220
Brain Hypothalamus	HYP	158	1,220
Brain Nucleus accumbens basal ganglia	NAB	183	1,391
Brain Putamen basal ganglia	PBG	155	1,427
Brain Spinal cord cervical c-1	SCC	116	1,335
Brain Substantia nigra	SUB	102	1,092
Total		1,990	19,632

[a]Use only European samples; [b]The genes located in major histocompatibility complex (MHC) region were removed. The MHC region occurs on chromosome 6, between the flanking genetic markers *MOG* and *COL11A2* (from 6p22.1 to 6p21.3 about 29Mb to 33Mb on the hg38 assembly).

Supplementary Table 2. Significant TWAS genes for PD.

Gene	Chr	Gene Start	Gene End	Tissue	Best eQTL	TWAS Z-score	TWAS *P*-value	Permutation *P*-value
LRRC37A2	chr17	46511511	46555650	HYP	rs17689918	-9.63	6.12E-22	0.0256
				NAB	rs17762769	-9.40	5.62E-21	0.0061
				ACC	rs17689471	-9.30	1.44E-20	0.0373
				SCC	rs1396862	-9.29	1.59E-20	0.0027
				HIP	rs17763086	-9.23	2.62E-20	0.1430
				CEH	rs8072451	-9.23	2.79E-20	0.0429
				PBG	rs1396862	-9.22	3.06E-20	0.0033
				COR	rs17689824	-9.22	3.07E-20	0.0241
				CBG	rs171440	-9.20	3.46E-20	0.0241
				AMY	rs171440	-9.20	3.52E-20	0.0044
				CER	rs4566211	-9.02	1.82E-19	0.0030
				FRO	rs4566211	-9.00	2.18E-19	0.0432
				SUB	rs199443	-8.34	7.44E-17	0.0015
LRRC37A	chr17	46292733	46337794	CER	rs17762769	-9.36	7.85E-21	0.0635
				SCC	rs17762769	-9.28	1.65E-20	0.0407
				CEH	rs199449	-7.70	1.40E-14	0.3158
				SUB	rs4277389	-7.57	3.65E-14	0.1188
				AMY	rs35732828	-4.99	6.06E-07	0.4140
MMRN1	chr4	89879532	89954629	FRO	rs3857059	9.34	9.49E-21	0.0916
				HIP	rs10021524	5.48	4.32E-08	0.0863
				SUB	rs1398946	5.43	5.51E-08	0.5217
				HYP	rs1442138	5.33	9.89E-08	0.1008
				CEH	rs7683789	4.98	6.33E-07	0.0415
ARL17A	chr17	46516702	46579682	PBG	rs17762769	-9.31	1.33E-20	0.0025
				AMY	rs17689471	-9.30	1.44E-20	0.0035
				NAB	rs199533	-9.12	7.74E-20	0.0075
				COR	rs199456	-8.95	3.48E-19	0.0043
				FRO	rs17689918	-8.85	8.41E-19	0.0063
				HYP	rs199536	-8.81	1.22E-18	0.0033
				CEH	rs8072451	-8.38	5.18E-17	0.0078
				CER	rs17689471	-8.38	5.20E-17	0.0158
				ACC	rs35937770	-7.84	4.55E-15	0.0119
				HIP	rs17689918	-7.18	6.83E-13	0.0577
				CBG	rs17762769	-6.84	8.02E-12	0.0254
PLEKHM1	chr17	45435900	45490749	CEH	rs17689918	8.94	3.76E-19	0.0001
				CER	rs171440	8.48	2.30E-17	0.0003
FMNL1	chr17	45221444	45247320	CER	rs17689471	8.65	4.95E-18	0.0007
				CEH	rs12449792	8.23	1.80E-16	0.0016
CD38	chr4	15778275	15853230	COR	rs1558451	-6.85	7.52E-12	0.0006
				PBG	rs13124087	-4.97	6.67E-07	0.0004
RNF40	chr16	30761745	30776307	CEH	rs7500719	-6.24	4.42E-10	0.1936

SPPL2C	chr17	45844835	45847072	CEH	rs199456	-6.00	1.98E-09	0.0541
				CER	rs3744760	-4.89	1.01E-06	0.0960
VKORC1	chr16	31090842	31095980	COR	rs8062719	5.95	2.66E-09	0.0176
CCDC189	chr16	30757423	30762710	COR	rs4889490	5.91	3.35E-09	0.0784
				NAB	rs1458201	5.59	2.23E-08	0.0569
GPNMB	chr7	23235967	23275108	AMY	rs6461687	5.84	5.31E-09	0.0031
				CER	rs199355	5.79	7.00E-09	0.0043
				PBG	rs6461687	5.76	8.47E-09	0.0095
				COR	rs156429	5.73	1.02E-08	0.0146
				CBG	rs2072369	5.61	2.02E-08	0.0036
				ACC	rs199355	5.55	2.87E-08	0.0149
				NAB	rs7796541	5.45	4.90E-08	0.0033
				HYP	rs7796541	5.00	5.66E-07	0.0039
NUPL2	chr7	23181827	23201011	CEH	rs7808488	5.69	1.24E-08	0.0096
				CER	rs7808488	5.65	1.65E-08	0.0146
MAP3K14	chr17	45263121	45317040	CER	rs9908905	5.51	3.52E-08	0.0001
ZSWIM7	chr17	15976560	15999717	CEH	rs4792721	-5.38	7.25E-08	0.0168
				FRO	rs2157991	5.20	2.04E-07	0.0625
				CER	rs1045599	-5.19	2.05E-07	0.0577
				CBG	rs758853	5.11	3.29E-07	0.0132
				NAB	rs9891938	5.03	4.80E-07	0.0800
				COR	rs178797	4.93	8.22E-07	0.0945
				ACC	rs2157991	4.89	1.01E-06	0.0225
GAK	chr4	849276	932373	CER	rs4974574	5.21	1.85E-07	0.0262
CENPV	chr17	16342534	16353656	FRO	rs8076864	4.99	6.07E-07	0.0001
CPLX1	chr4	784957	826198	CEH	rs1134921	-4.98	6.36E-07	0.0531

Note: HYP, Brain Hypothalamus; NAB, Brain Nucleus accumbens basal ganglia; ACC, Brain Anterior cingulate cortex BA24; SCC, Brain Spinal cord cervical c-1; HIP, Brain Hippocampus; CEH, Brain Cerebellar Hemisphere; PBG, Brain Putamen basal ganglia; COR, Brain Cortex; CBG, Brain Caudate basal ganglia; AMY, Brain Amygdala; CER, Brain Cerebellum; FRO, Brain Frontal Cortex BA9; SUB, Brain Substantia nigra.

Supplementary Table 3. Replication results of the conditionally independent genes.

Gene	Tissue	TWAS Z-score	TWAS P-value
**LRRC37A2**	HIP	-6.55	5.86E-11
	HYP	-6.41	1.41E-10
	NAB	-6.23	4.57E-10
	SUB	-6.23	4.61E-10
	ACC	-6.18	6.29E-10
	CEH	-6.17	6.70E-10
	CER	-6.11	9.85E-10
	PBG	-6.09	1.11E-09
	SCC	-6.08	1.19E-09
	CBG	-6.08	1.21E-09
	AMY	-6.08	1.23E-09
	COR	-6.01	1.80E-09
	FRO	-5.83	5.66E-09
**MMRN1**	FRO	4.96	7.16E-07
	CEH	3.93	8.39E-05
	HIP	3.91	9.30E-05
	HYP	3.53	4.10E-04
	PBG	3.04	2.34E-03
	SUB	NA	NA
CD38	CEH	-3.12	1.79E-03
	PBG	-2.24	0.025
	COR	-2.17	0.030
	CER	-1.72	0.085
	FRO	-0.7	0.483
RNF40	CEH	-3.06	2.20E-03
**GPNMB**	ACC	3.82	1.32E-04
	PBG	3.78	1.54E-04
	CER	3.78	1.56E-04
	AMY	3.72	2.00E-04
	COR	3.67	2.46E-04
	HIP	3.63	2.85E-04
	CBG	3.44	5.72E-04
	NAB	3.05	2.30E-03
	HYP	2.5	0.012
**ZSWIM7**	AMY	-3.44	5.74E-04
	CEH	-0.76	0.447
	CER	-0.66	0.508
	FRO	0.54	0.590
	ACC	0.46	0.648
	NAB	0.35	0.723
	HYP	0.35	0.726
	CBG	0.3	0.765

	COR	NA	NA
GAK	CER	2.08	0.037
**CPLX1**	CEH	-4.39	1.14E-05
	CER	-0.26	0.792

Supplementary Table 4. The 122 GWAS summary data from other public resources.

Trait	Type	Sample Size (Cases)	URL/pmid
Life satisfaction (univariate)	Activities	80852	30643256
Types of physical activity in last 4 weeks: Heavy DIY	Activities	384450 (165465)	http://atlas.ctglab.nl/ukb2_sumstats
Weekly usage of mobile phone in last 3 months	Activities	322862	http://atlas.ctglab.nl/ukb2_sumstats
Duration walking for pleasure	Activities	323744	https://cnsgenomics.com/software/gcta/#UKBiobankGWASresults
Health satisfaction	Activities	150001	https://cnsgenomics.com/software/gcta/#UKBiobankGWASresults
Mineral and other dietary supplements: Glucosamine	Activities	454825 (88339)	https://cnsgenomics.com/software/gcta/#UKBiobankGWASresults
Types of transport used (excluding work): Walk	Activities	453928 (222804)	https://cnsgenomics.com/software/gcta/#UKBiobankGWASresults
Usual walking pace	Activities	453401	https://cnsgenomics.com/software/gcta/#UKBiobankGWASresults
Vitamin and mineral supplements: Vitamin C	Activities	453791 (39182)	https://cnsgenomics.com/software/gcta/#UKBiobankGWASresults
Overall health rating	Activities	454314	https://cnsgenomics.com/software/gcta/#UKBiobankGWASresults
Non-cancer illness code, self-reported: hypertension	Cardiovascular	289307	http://atlas.ctglab.nl/ukb2_sumstats
Pulse rate (automated reading)	Cardiovascular	361411	http://atlas.ctglab.nl/ukb2_sumstats
Hypertension	Cardiovascular	361141 (93560)	https://www.dropbox.com
Diagnosed by doctor: High blood pressure	Cardiovascular	455303 (122620)	https://cnsgenomics.com/software/gcta/#UKBiobankGWASresults
Systolic Blood Pressure (automated reading)	Cardiovascular	361402	https://atlas.ctglab.nl
High blood pressure	Cardiovascular	458554 (144793)	ftp://ftp.ebi.ac.uk/pub/databases/gwas/summary_statistics
Resting heart rate	Cardiovascular	24218	ftp://ftp.ebi.ac.uk/pub/databases/gwas/summary_statistics
Fluid intelligence test - Fluid intelligence score	Cognitive	99739	http://atlas.ctglab.nl/ukb2_sumstats
Intelligence	Cognitive	269867	https://ctg.cncr.nl/documents/p1651/SavageJansen_IntMeta_sumstats.zip
Verbal-numerical reasoning	Cognitive	168033	29844566
Cognitive performance	Cognitive	257828	https://www.thessgac.org/data
Fluid intelligence score	Cognitive	146808	https://cnsgenomics.com/software/gcta/#UKBiobankGWASresults
Maximum digits remembered correctly	Cognitive	89799	https://atlas.ctglab.nl
Mean time to correctly identify matches	Cognitive	383748	https://atlas.ctglab.nl
Lymphocyte count (three-way meta)	Immunological	171643	https://cnsgenomics.com/software/gcta/#UKBiobankGWASresults
Lymphocyte percentage of white cells (three-way meta)	Immunological	171748	https://cnsgenomics.com/software/gcta/#UKBiobankGWASresults
Mean corpuscular volume (three-way meta)	Immunological	172433	https://cnsgenomics.com/software/gcta/#UKBiobankGWASresults
Neutrophil percentage of white cells (three-way meta)	Immunological	171542	https://cnsgenomics.com/software/gcta/#UKBiobankGWASresults
Granulocyte count (three-way meta)	Immunological	169822	http://data.qld.edu.au/public/
Lymphocyte count (two-way meta)	Immunological	132452	ftp://ftp.sanger.ac.uk/pub/project/humgen/summary_statistics

Lymphocyte percentage of white cells (two-way meta)	Immunological	132570	ftp://ftp.sanger.ac.uk/pub/project/humgen/summary_statistics
Mean corpuscular hemoglobin (three-way meta)	Immunological	172332	ftp://ftp.sanger.ac.uk/pub/project/humgen/summary_statistics
Mean corpuscular hemoglobin (two-way meta)	Immunological	132224	ftp://ftp.sanger.ac.uk/pub/project/humgen/summary_statistics
Mean corpuscular volume (two-way meta)	Immunological	132353	ftp://ftp.sanger.ac.uk/pub/project/humgen/summary_statistics
Mean platelet volume (two-way meta)	Immunological	127230	ftp://ftp.sanger.ac.uk/pub/project/humgen/summary_statistics
Neutrophil count (three-way meta)	Immunological	170702	ftp://ftp.sanger.ac.uk/pub/project/humgen/summary_statistics
Neutrophil percentage of white cells (two-way meta)	Immunological	132352	ftp://ftp.sanger.ac.uk/pub/project/humgen/summary_statistics/
Blood urea nitrogen	Metabolic	243031	31152163
Hip circumference	Metabolic	385887	http://atlas.ctglab.nl/ukb2_sumstats
Impedance of leg (left)	Metabolic	379807	http://atlas.ctglab.nl/ukb2_sumstats
Impedance of leg (right)	Metabolic	379813	http://atlas.ctglab.nl/ukb2_sumstats
Leg fat percentage (right)	Metabolic	379806	http://atlas.ctglab.nl/ukb2_sumstats
Waist-hip ratio	Metabolic	93480	http://portals.broadinstitute.org/collaboration/giant/index.php/Main_Page
Waist-hip ratio (adjusted for BMI)	Metabolic	210086	http://portals.broadinstitute.org/collaboration/giant/index.php/Main_Page
Waist-hip ratio (adjusted for BMI, female)	Metabolic	116742	http://portals.broadinstitute.org/collaboration/giant/index.php/Main_Page
Body Mass Index	Metabolic	322154	http://portals.broadinstitute.org/collaboration/giant/index.php/Main_Page
Body Mass Index (female)	Metabolic	171977	http://portals.broadinstitute.org/collaboration/giant/index.php/Main_Page
Comparative body size at age 10	Metabolic	448349	https://cnsgenomics.com/software/gcta/#UKBiobankGWASresults
Arm fat-free mass (left)	Metabolic	379653	http://atlas.ctglab.nl/ukb2_sumstats
Arm fat mass (left)	Metabolic	379663	http://atlas.ctglab.nl/ukb2_sumstats
Arm fat percentage (left)	Metabolic	379663	http://atlas.ctglab.nl/ukb2_sumstats
Arm fat percentage (right)	Metabolic	379663	http://atlas.ctglab.nl/ukb2_sumstats
Body fat percentage	Metabolic	379615	http://atlas.ctglab.nl/ukb2_sumstats
Impedance of arm (left)	Metabolic	379803	http://atlas.ctglab.nl/ukb2_sumstats
Leg fat-free mass (left)	Metabolic	379766	http://atlas.ctglab.nl/ukb2_sumstats
Leg fat-free mass (right)	Metabolic	379793	http://atlas.ctglab.nl/ukb2_sumstats
Leg fat mass (left)	Metabolic	379783	http://atlas.ctglab.nl/ukb2_sumstats
Leg fat mass (right)	Metabolic	379802	http://atlas.ctglab.nl/ukb2_sumstats
Leg fat percentage (left)	Metabolic	379786	http://atlas.ctglab.nl/ukb2_sumstats
Leg predicted mass (right)	Metabolic	379793	http://atlas.ctglab.nl/ukb2_sumstats
Trunk fat mass	Metabolic	379578	http://atlas.ctglab.nl/ukb2_sumstats
Trunk fat percentage	Metabolic	379600	http://atlas.ctglab.nl/ukb2_sumstats

Whole body fat-free mass	Metabolic	379804	http://atlas.ctglab.nl/ukb2_sumstats
Whole body water mass	Metabolic	379835	http://atlas.ctglab.nl/ukb2_sumstats
Waist circumference	Metabolic	455545	https://cnsgenomics.com/software/gcta/#UKBiobankGWASresults
C-reactive protein	Metabolic	28520	ftp://ftp.ebi.ac.uk/pub/databases/gwas/summary_statistics
Cancer (diagnosed by doctor)	Other	385231	http://atlas.ctglab.nl/ukb2_sumstats
Chest pain or discomfort when walking uphill or hurrying	Other	48190	http://atlas.ctglab.nl/ukb2_sumstats
Educational attainment	Other	95427	https://www.thessgac.org/data
FEV1	Other	400102	http://data.qld.edu.au/public/Q1031/UKB_impute_v1/3063.v1.fastGWA.gz
Male pattern baldness (BOLT LMM non-infinitesimal mixed model)	Other	205327	http://cnsgenomics.com/data/mpb/mpb_bolt_lmm_aut_x.tab.zip
Open-angle glaucoma	Other	22795	https://storage.googleapis.com/finngen-public-data-r2/summary_stats
Age at cancer diagnosis	Other	73638	https://cnsgenomics.com/software/gcta/#UKBiobankGWASresults
Bread type: White	Other	440084 (115996)	https://cnsgenomics.com/software/gcta/#UKBiobankGWASresults
Cereal intake	Other	435601	https://cnsgenomics.com/software/gcta/#UKBiobankGWASresults
Cheese intake	Other	64001	https://cnsgenomics.com/software/gcta/#UKBiobankGWASresults
Crohn's Disease	Other	69268 (22575)	https://www.ibdgenetics.org/downloads.html
Fresh fruit intake	Other	440074	https://cnsgenomics.com/software/gcta/#UKBiobankGWASresults
Friendships satisfaction	Other	148842	https://cnsgenomics.com/software/gcta/#UKBiobankGWASresults
Hair colour (natural, before greying): Blonde	Other	385603 (43319)	https://atlas.ctglab.nl
FVC	Other	400102	http://data.qld.edu.au/public/Q1031/UKB_impute_v1/3062.v1.fastGWA.gz
Age first had sexual intercourse	Other	339614	https://atlas.ctglab.nl
Job involves mainly walking or standing	Other	259397	https://cnsgenomics.com/software/gcta/#UKBiobankGWASresults
Hair/balding pattern: Pattern 1	Other	176380 (56514)	https://atlas.ctglab.nl
Hair/balding pattern: Pattern 4	Other	176380 (32228)	https://atlas.ctglab.nl
Never eat eggs, dairy, wheat, sugar: Wheat products	Other	384986 (10236)	https://atlas.ctglab.nl
Number of self-reported cancers	Other	456269	https://cnsgenomics.com/software/gcta/#UKBiobankGWASresults
Type 2 Diabetes	Other	898130 (74124)	http://diagram-consortium.org/downloads.html
Ulcerative colitis	Other	72647 (20417)	https://www.ibdgenetics.org/downloads.html
Oily fish intake	Other	453893	https://cnsgenomics.com/software/gcta/#UKBiobankGWASresults
Long-standing illness, disability or infirmity	Other	377498 (122262)	https://atlas.ctglab.nl
Bilateral oophorectomy (both ovaries removed) (female)	Other	205791 (16654)	https://atlas.ctglab.nl
Education - Qualifications	Other	318526	https://atlas.ctglab.nl

FEV1/FVC ratio	Other	79055	ftp://ftp.ebi.ac.uk/pub/databases/gwas/summary_statistics
Male pattern baldness (BOLT LMM infinitesimal mixed model)	Other	205327	http://cnsgenomics.com/data/mpb/mpb_bolt_lmm_aut_x.tab.zip
Offspring birthweight (maternal)	Other	86577	http://mccarthy.well.ox.ac.uk/publications
PEF	Other	24218	ftp://ftp.ebi.ac.uk/pub/databases/gwas/summary_statistics
Gestational weight gain (maternal, total)	Other	10555	http://egg-consortium.org/downloads
Ease of getting up in the morning	Psychiatric	345552 (91820)	https://ctg.cncr.nl
General happiness	Psychiatric	126132	http://atlas.ctglab.nl/ukb2_sumstats
Well-being spectrum	Psychiatric	2311184	30643256
Anxiety - Recent inability to stop or control worying	Psychiatric	126300	https://atlas.ctglab.nl
Current tobacco smoking	Psychiatric	455825	https://cnsgenomics.com/software/gcta/#UKBiobankGWASresults
Frequency of drinking alcohol	Psychiatric	147581	https://cnsgenomics.com/software/gcta/#UKBiobankGWASresults
Getting up in morning	Psychiatric	455090	https://cnsgenomics.com/software/gcta/#UKBiobankGWASresults
Happiness	Psychiatric	146970	https://cnsgenomics.com/software/gcta/#UKBiobankGWASresults
Been in serious accident believed to be life-threatening	Psychiatric	126665 (12432)	https://atlas.ctglab.nl
Worrier / anxious feelings	Psychiatric	444404 (251982)	https://cnsgenomics.com/software/gcta/#UKBiobankGWASresults
Sleep duration (mean)	Psychiatric	384317	https://ctg.cncr.nl/documents/p1651/Sleepdur_sumstats_Jansenetal.txt.gz
Neuroticism	Psychiatric	329821	http://www.psy.ed.ac.uk/ccace/downloads/Luciano_2017.zip
Anorexia nervosa	Psychiatric	14477 (3495)	https://www.med.unc.edu/pgc/results-and-downloads
Heel bone mineral density	Skeletal	394929	http://atlas.ctglab.nl/ukb2_sumstats
Comparative height size at age 10	Skeletal	448718	https://cnsgenomics.com/software/gcta/#UKBiobankGWASresults
Non-cancer illness code, self-reported: osteoarthritis	Skeletal	361141 (30046)	https://www.dropbox.com
Sitting height	Skeletal	454934	https://cnsgenomics.com/software/gcta/#UKBiobankGWASresults
Standing height	Skeletal	452264	http://geneatlas.roslin.ed.ac.uk/downloads/
Total body BMD	Skeletal	66628	http://www.gefos.org/?q=documents
Birth length	Skeletal	28459	http://egg-consortium.org/downloads/EGG-GWAS-BL.txt.gz
Estimated BMD	Skeletal	142487	http://www.gefos.org/sites/default/files/
Height	Skeletal	49796	ftp://ftp.ebi.ac.uk/pub/databases/gwas/summary_statistics
Osteoarthritis	Skeletal	455221 (77052)	ftp://ftp.ebi.ac.uk/pub/databases/gwas/summary_statistics
Osteoarthritis of hip	Skeletal	393873 (15704)	ftp://ftp.ebi.ac.uk/pub/databases/gwas/summary_statistics

Supplementary Table 5. The significant TWAS results of the genes we identified in the Kia's paper.

[a]Gene	Chr	[b]P0	P1	Z-score	*P*-value	FDR
MMRN1	4	89879532	89954629	10.21	1.86×10-24	2.52×10-21
CD38	4	15778275	15853230	-9.60	7.66×10-22	8.30×10-19
GPNMB	7	23235967	23275108	8.51	1.69×10-17	1.53×10-14
NUPL2	7	23181827	23201011	8.35	7.02×10-17	5.11×10-14
VKORC1	16	31090842	31095980	5.34	9.42×10-8	2.22×10-5
CENPV	17	16342534	16353656	3.63	2.86×10-4	0.0158
RNF40	16	30761745	30776307	-3.32	8.94×10-4	0.0381

[a]Genes are ordered by the TWAS *P*-value in CMC dorsolateral prefrontal cortex reported by Kia and colleagues; [b]Gene positions are assigned to the CRCh38 human reference genome assembly. Note: Chr, chromosome; FDR, false discovery rate.

Supplementary Figure 1. Heatmap of the associations between each SNP in the final models of the 8 identified genes and the top five phenotypes (excluding PD) identified via pheWAS.

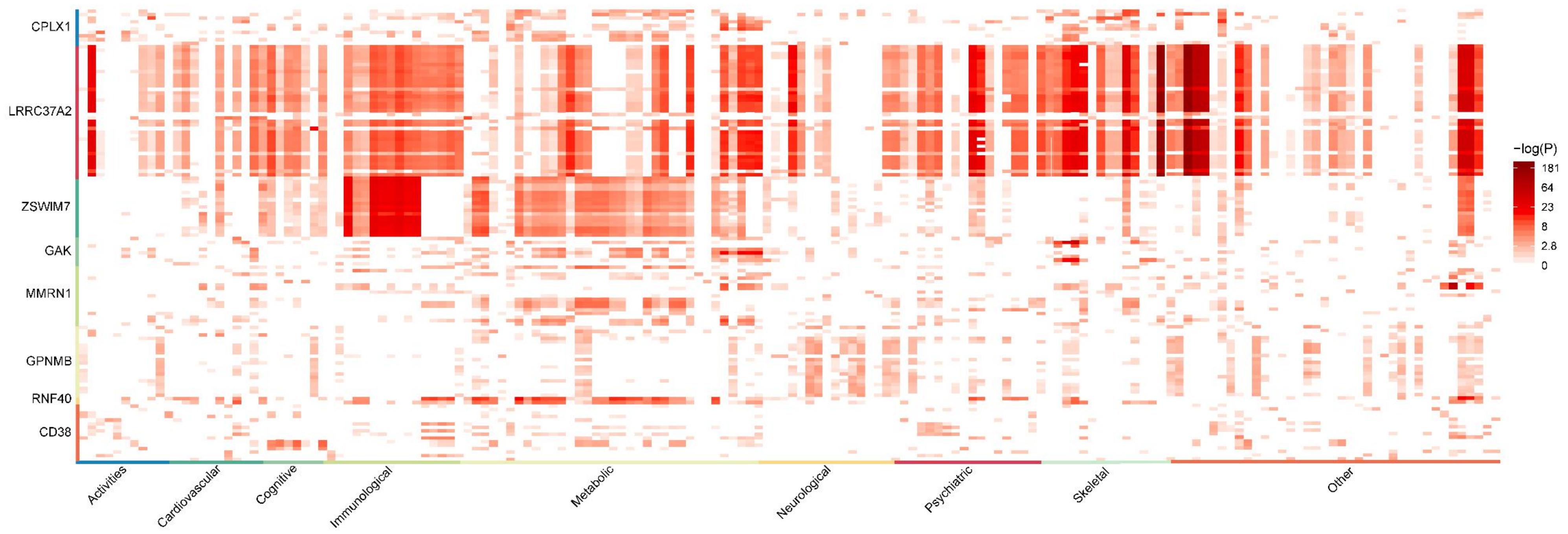

Supplement: Supplementary file 1 — Supplementary information. [file 41531_2021_221_MOESM1_ESM.pdf]
