# Supplementary material for: A transcriptome-wide association study identifies susceptibility genes for Parkinson’s disease
Source: NPJ Parkinsons Dis. 2021 Sep 9;7:79. doi: 10.1038/s41531-021-00221-7 (PMC8429416; doi:10.1038/s41531-021-00221-7)
Supplement: Supplementary file 2 — Reporting summary. [file 41531_2021_221_MOESM2_ESM.pdf]

nature research

Corresponding author(s): Xiao-Ye Wang, Liang Li, Xiao-Song Li

Last updated by author(s): Jul 30, 2021

Reporting Summary

Nature Research wishes to improve the reproducibility of the work that we publish. This form provides structure for consistency and transparency in reporting. For further information on Nature Research policies, see our Editorial Policies and the Editorial Policy Checklist.

Statistics

For all statistical analyses, confirm that the following items are present in the figure legend, table legend, main text, or Methods section.

n/a	Confirmed	
☒	☐	The exact sample size (*n*) for each experimental group/condition, given as a discrete number and unit of measurement
☒	☐	A statement on whether measurements were taken from distinct samples or whether the same sample was measured repeatedly
☒	☐	The statistical test(s) used AND whether they are one- or two-sided<br>*Only common tests should be described solely by name; describe more complex techniques in the Methods section.*
☒	☐	A description of all covariates tested
☐	☒	A description of any assumptions or corrections, such as tests of normality and adjustment for multiple comparisons
☒	☐	A full description of the statistical parameters including central tendency (e.g. means) or other basic estimates (e.g. regression coefficient) AND variation (e.g. standard deviation) or associated estimates of uncertainty (e.g. confidence intervals)
☐	☒	For null hypothesis testing, the test statistic (e.g. *F*, *t*, *r*) with confidence intervals, effect sizes, degrees of freedom and *P* value noted<br>*Give P values as exact values whenever suitable.*
☒	☐	For Bayesian analysis, information on the choice of priors and Markov chain Monte Carlo settings
☒	☐	For hierarchical and complex designs, identification of the appropriate level for tests and full reporting of outcomes
☒	☐	Estimates of effect sizes (e.g. Cohen's *d*, Pearson's *r*), indicating how they were calculated

Our web collection on statistics for biologists contains articles on many of the points above.

Software and code

Policy information about availability of computer code

Data collection	No software was used to collect data.
Data analysis	glmnet (2.0-16) R package was used to built lasso and elastic net models.<br>FUSION (http://gusevlab.org/projects/fusion/) was used to perform TWAS, permutation, and conditional tests.<br>LDSC (https://github.com/bulik/ldsc) was used to perform partitioned heritability estimation.<br>DAVID (v6.8) was used to perform gene set enrichment analyses.<br>Custom code (https://github.com/studentyaoshi/etwas) was used to generate gene expression prediction models.

For manuscripts utilizing custom algorithms or software that are central to the research but not yet described in published literature, software must be made available to editors and reviewers. We strongly encourage code deposition in a community repository (e.g. GitHub). See the Nature Research guidelines for submitting code & software for further information.

Data

Policy information about availability of data

All manuscripts must include a data availability statement. This statement should provide the following information, where applicable:

- Accession codes, unique identifiers, or web links for publicly available datasets
- A list of figures that have associated raw data
- A description of any restrictions on data availability

The data that support the findings of this study are available from the corresponding author upon reasonable request.

Field-specific reporting

Please select the one below that is the best fit for your research. If you are not sure, read the appropriate sections before making your selection.

☒ Life sciences ☐ Behavioural & social sciences ☐ Ecological, evolutionary & environmental sciences

For a reference copy of the document with all sections, see nature.com/documents/nr-reporting-summary-flat.pdf

Life sciences study design

All studies must disclose on these points even when the disclosure is negative.

Sample size	No statistical method was used to determine sample size for PD summary statistic and eqtl data. We used the GWAS summary statistic and eQTL data with the largest known sample size.
Data exclusions	No data were excluded from the analysis.
Replication	Another GWAS summary statistic of PD, comprising 4,238 PD cases, and 4,239 controls, was used as the replication data of this study. We found 46 reference models for the identified eight genes, and five genes significantly associated with PD in at least one brain tissue.
Randomization	It is not relevant to this study since we only used the PD GWAS summary statistic and the eqtl data without phenotypes.
Blinding	Blinding was not possible since we only used the PD GWAS summary statistic and the eqtl data without phenotypes.

Reporting for specific materials, systems and methods

We require information from authors about some types of materials, experimental systems and methods used in many studies. Here, indicate whether each material, system or method listed is relevant to your study. If you are not sure if a list item applies to your research, read the appropriate section before selecting a response.

Materials & experimental systems

n/a	Involved in the study	
☒	☐	Antibodies
☒	☐	Eukaryotic cell lines
☒	☐	Palaeontology and archaeology
☒	☐	Animals and other organisms
☒	☐	Human research participants
☒	☐	Clinical data
☒	☐	Dual use research of concern

Methods

n/a	Involved in the study	
☒	☐	ChIP-seq
☒	☐	Flow cytometry
☒	☐	MRI-based neuroimaging
